# Robust prostate cancer risk stratification from unregistered mpMRI via learned cross-modal correspondence

**DOI:** 10.3389/fonc.2025.1700447

**Published:** 2026-01-29

**Authors:** Hanying Gong, Jie Luo, Shufan Mao, Yanchen Gong, Yu Lu, Jian Ding, Xiang Zhu

**Affiliations:** 1 Department of Radiology, The First Hospital of Jiaxing, The Affiliated Hospital of Jiaxing University, Jiaxing, China; 2 Department of Health Care, Jinling Hospital, Medical School of Nanjing University, Nanjing, China; 3 Perception Vision Medical Technologies Co Ltd, Guangzhou, China; 4 Jiaxing University Master Degree Cultivation Base, Zhejiang Chinese Medical University, Jiaxing, China

**Keywords:** deep learning, feature fusion, image registration, multiparametric MRI, optimal transport, prostate cancer

## Abstract

**Background and objective:**

Accurate prostate cancer risk stratification benefits from the fusion of T2-weighted (T2WI) and Apparent Diffusion Coefficient (ADC) MRI. However, patient motion and imaging distortions frequently cause spatial misalignments between these sequences. While radiologists compensate for this via subjective cognitive fusion, the process introduces inter-reader variability and can be particularly challenging in equivocal cases. Conventional fusion models are even more vulnerable, as they require perfect image registration, making them brittle in real-world clinical scenarios. We aimed to develop and validate a deep learning framework that overcomes these limitations by robustly fusing unregistered mpMRI data.

**Methods:**

We retrospectively analyzed a cohort of 300 consecutive men (mean age, 71.5 ± 7.6 years) who underwent pre-biopsy prostate mpMRI at our institution between January 2021 and May 2023. All included patients had pathologically confirmed prostate cancer, with high-risk prostate cancer, as defined by NCCN guidelines, present in 184 of 300 cases (61.3%). The dataset was partitioned chronologically into a development cohort (n=250) for 5-fold cross-validation and a temporal test cohort (n=50) for independent evaluation. We developed Cross-Modal Optimal Transport Fusion (CMOT-Fusion), a deep learning framework that learns to identify and match diagnostically relevant regions between misaligned T2WI and ADC images. This approach enables robust multimodal fusion without requiring an explicit image registration step.

**Results:**

For discriminating NCCN high-risk versus low/intermediate-risk disease among pathologically confirmed prostate cancer cases, CMOT-Fusion achieved a mean Area Under the Curve (AUC) of 0.849 ± 0.034 in 5-fold cross-validation, outperforming single-modality baselines and conventional fusion methods. On an independent test set, the model’s performance remained robust, with an ensemble AUC of 0.824 (95% CI: 0.694–0.930; ensemble probability computed as the mean of the five fold-specific model probabilities per patient). As a cohort-specific clinical reference based on routine radiology suspicion scoring, PI-RADS v2.1 achieved an AUC of 0.839 (95% CI: 0.726–0.930) on the same test cohort.

**Conclusion:**

Our results demonstrate that learning a direct correspondence between unregistered mpMRI sequences significantly improves prostate cancer risk stratification. The proposed CMOT-Fusion framework offers a robust solution to the common clinical problem of inter-sequence misalignment, potentially enhancing diagnostic reliability and streamlining clinical workflows by removing the need for a separate image registration step. Given the single-center retrospective design and the small independent test cohort, these findings should be considered exploratory and warrant multi-center prospective validation.

## 1 Introduction

Prostate cancer is a leading cause of cancer-related mortality in men globally and represents a significant health concern for aging populations due to its rising incidence (1–3). Accurate prostate cancer risk stratification is critical to the selection of treatment options for patients, which has a direct impact on prognosis. This process is guided by frameworks like the National Comprehensive Cancer Network (NCCN) guidelines, which classify risk based on clinical and pathological factors (4). Multiparametric MRI (mpMRI) has become a cornerstone of the diagnostic pathway, helping to detect clinically significant disease and guide biopsies (5). The effective fusion of its key sequences—T2-weighted (T2WI) for anatomical detail and Apparent Diffusion Coefficient (ADC) maps for tissue cellularity—is fundamental for accurate non-invasive assessment, yet remains a challenge (6, 7).

Beyond diagnosis, risk stratification has direct implications for clinical management. In routine practice, patients categorized as low or intermediate risk may be candidates for active surveillance or less intensive management, whereas high-risk disease typically prompts treatment escalation (e.g., definitive local therapy and/or multimodal strategies) to reduce the risk of progression and metastasis (4). Because these decisions are high-stakes and often time-sensitive, there is a clear need for imaging-derived assessments that reliably support NCCN-level risk grouping. mpMRI contributes to this decision-making by characterizing lesion morphology and diffusion restriction; however, when key sequences (T2WI and ADC) are misaligned or degraded by artifacts, both human interpretation and automated methods may become less consistent, motivating robust multimodal fusion strategies that do not depend on explicit registration.

Despite its diagnostic power, the clinical utility of mpMRI is often hampered by spatial misalignments between T2WI and ADC sequences. Such discrepancies are common, arising from patient motion, involuntary soft-tissue deformations such as bowel peristalsis, and sequence-specific geometric distortions, especially between T2WI and echo-planar DWI acquisitions (8, 9). Radiologists learn to compensate for these shifts by cognitively aligning anatomical landmarks, but this manual process is time-consuming and subjective 10, 11). This subjectivity contributes to significant inter-reader variability in mpMRI interpretation (e.g., *κ* ≈ 0.46–0.61 in PI-RADS scoring), which can impact the reliability of inputs for NCCN-level risk stratification (12). This dependence on a manual, subjective skill presents a major bottleneck to achieving reproducible and scalable risk assessment.

Computer-aided diagnosis (CAD) systems have emerged as a potential solution, but they face the challenge of handling inter-sequence misalignment (9). The conventional approach involves image registration as a preprocessing step. However, this strategy has major limitations: complex deformable algorithms may introduce artifacts, while rigid registration fails to correct for local deformations (8, 13). Furthermore, the downstream impact of registration errors on diagnostic performance is often unevaluated, making it unclear whether such preprocessing is beneficial (14). Alternative strategies that bypass registration often depend on strict acquisition protocols that are insufficient to prevent misalignment in practice (15). As a result, many existing CAD systems remain vulnerable to spatial inaccuracies, which limits their clinical robustness (16).

To overcome these challenges, we propose Cross-Modal Optimal Transport Fusion (CMOT-Fusion), a deep learning framework that robustly fuses information from unregistered T2WI and ADC images. Rather than depending on a separate, imperfect geometric alignment step, our model learns a direct functional correspondence between modalities. This mapping is guided by a cost function that is learned end-to-end to optimize the clinical task of NCCN risk stratification (high-risk versus low/intermediate-risk) among prostate cancer cases. By targeting the diagnostic objective instead of an intermediate registration task, CMOT-Fusion provides a more direct and robust solution. This paper introduces the methodology, presents a rigorous validation on a clinical cohort, and demonstrates its superior performance compared to conventional approaches.

## 2 Materials and methods

Key components and configuration details of the proposed CMOT-Fusion architecture are summarized in **Table 1**.

**Table 1:** CMOT-fusion model architecture: key components and configuration.

Component	Configuration	Function
Data Input & Preprocessing		
Patch Extraction	K=32 patches, 32×32×8 voxels	Z-score normalized, random sampling (training)
Input Modalities	T2WI and ADC volumes	Preprocessed, resampled to common space
Feature Encoders		
Architecture	3D ResNet-18, BasicBlock [2,2,2,2]	Twin encoders for modality-specific features
Output	256-dim embeddings, BatchNorm + scaling	Normalized feature vectors per patch
Cost Network & Optimal Transport		
Cost Network	MLP: 512→512→512→1, ReLU, Dropout(0.3)	Learns task-specific transport costs $C_{ij}$
Sinkhorn Solver	α=0.5, 30 iterations, log-domain	Computes OT plan $\pi^{*}$ with uniform marginals
Fusion & Classification		
Feature Fusion	Barycentric projection + addition	ADC→T2WI space mapping, LayerNorm
Classification	MLP: 256→256→classes, Dropout(0.3)	Average pooling → final predictions

### 2.1 Patient cohort and reference standard

**Figure 1:**
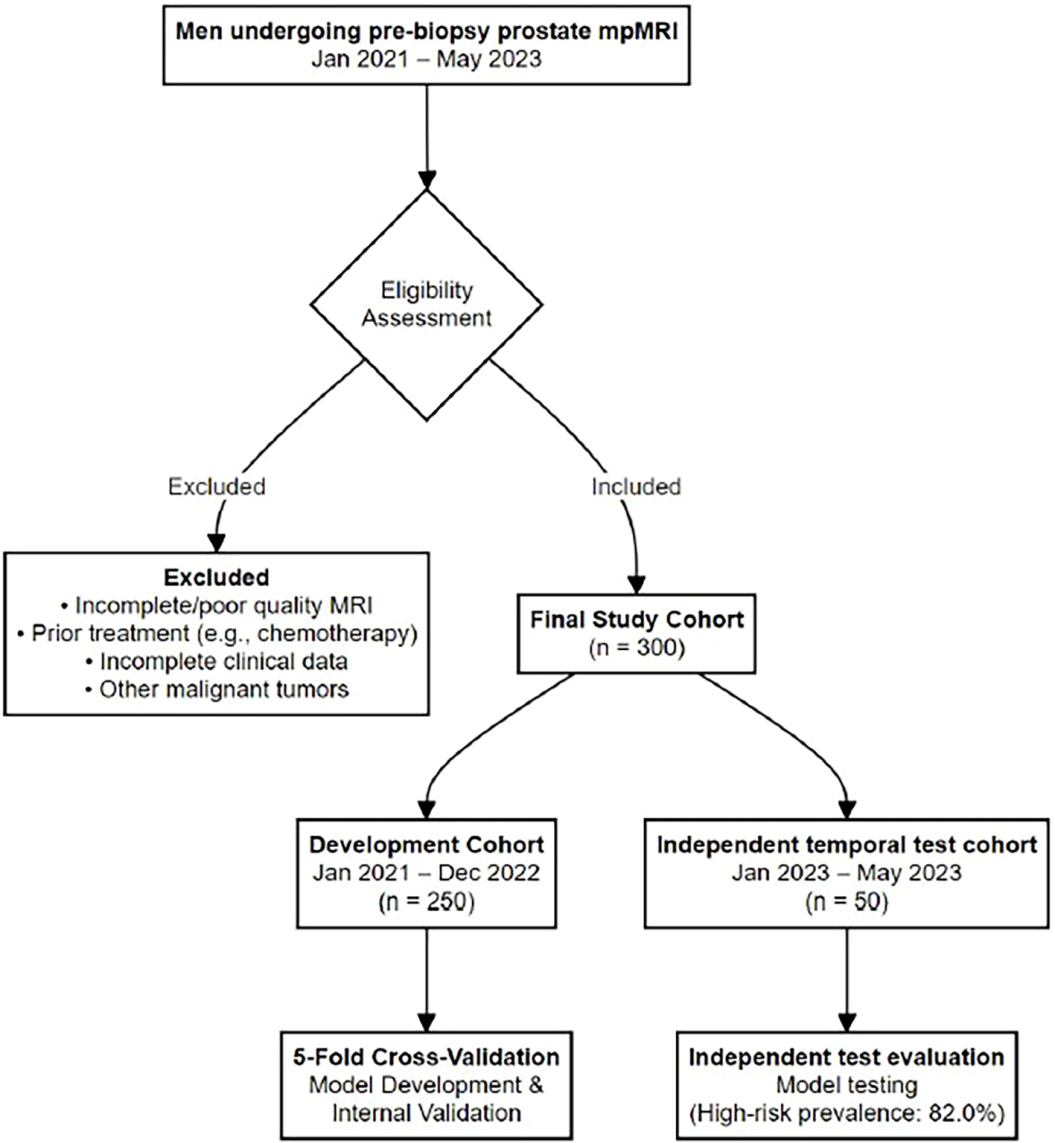
Patient selection flow diagram (CONSORT-style). This diagram summarizes the inclusion and exclusion criteria, the final cohort size (*n* = 300), and the temporal split into a development cohort (*n* = 250) and an independent temporal test cohort (*n* = 50).

This retrospective study was approved by the Institutional Review Board of The First Hospital of Jiaxing (approval #2025-LP-563) and complied with the Declaration of Helsinki; the requirement for written informed consent was waived. We analyzed a cohort of 300 male patients who underwent multiparametric prostate MRI (mpMRI) at our institution between January 2021 and May 2023. Pathology results, which served as the reference standard, were obtained within two months of the MRI examination from transrectal ultrasound-guided biopsy, transurethral resection of the prostate, or radical prostatectomy specimens. Specimens were graded by a genitourinary pathologist according to 2014 International Society of Urological Pathology (ISUP) recommendations (17). Inclusion criteria were: (1) availability of pathology results within two months of the MRI examination; (2) clearly defined prostate lesions on T2WI and ADC images; and (3) no contraindications for MRI. Exclusion criteria were: (1) incomplete or poor quality MRI images; (2) prior treatment such as chemotherapy before the MRI; (3) incomplete clinical data; or (4) presence of other malignant tumors. All included patients had pathologically confirmed prostate cancer; no benign/no-cancer cases were included. The patient selection process is illustrated in **Figure 1**.

The dataset was temporally partitioned to create a development cohort (N, 250, scans from January 2021 to December 2022) for model training and internal validation, and an independent test cohort (N, 50, scans from January 2023 to May 2023) to simulate prospective deployment, following established methodological recommendations (18). The primary outcome was NCCN risk stratification among prostate cancer cases (high-risk versus low/intermediate-risk), defined based on NCCN guidelines as the presence of PSA ≥ 20 ng/mL, a Gleason Score *>* 7, or clinical stage T3a-T4 (4). Patients not meeting these criteria were categorized into a combined low/intermediate-risk group. For model development, we performed a 5-fold patient-level stratified cross-validation on the development cohort. The independent test cohort was held out for the final, unbiased evaluation of the trained models.

### 2.2 Patient demographics and clinical characteristics

The development cohort (n=250; mean age, 71.7 ± 7.5 years) and test cohort (n=50; mean age, 70.0 ± 8.0 years) reflect a chronological split (development: January 2021–December 2022; test: January 2023–May 2023). The prevalence of high-risk prostate cancer was 143/250 (57.2%) in the development set and 41/50 (82.0%) in the test set. Original diagnostic PI-RADS scores were determined by two board-certified abdominal radiologists (each with over 10 years of prostate MRI experience), with disagreements resolved by consensus. To quantify cohort differences in baseline variables, we performed two-sided nonparametric comparisons (Mann–Whitney U). Age did not differ significantly between cohorts (*p* = 0.254), whereas PSA and PI-RADS were higher in the independent test cohort (PSA *p* = 0.0119; PI-RADS *p* = 0.0150). Because PSA was highly skewed with extreme outliers, we additionally report robust summaries (median [IQR]) alongside mean ± SD. Detailed demographic, clinical, and imaging characteristics for both cohorts are summarized in **Table 2**.

**Table 2:** Detailed clinical and technical characteristics of study cohorts.

Characteristic	Development cohort	External test cohort	Overall	p -value^‡^
Demographics				
Number of patients	250	50	300	–
Age (years), mean ± SD [range]	71.7 ± 7.5 [41-90]	70.0 ± 8.0 [49-84]	71.5 ± 7.6 [41-90]	blue0.254
Clinical Parameters				
PSA level (ng/ml), mean ± SD [range]	101.2 ± 324.8 [0.0-2583.3] blue(n=242)	258.5 ± 903.6 [4.0-5266.0] blue(n=49)	127.7 ± 475.7 [0.0-5266.0] blue(n=291)	–
PSA level (ng/ml), median [IQR]	15.0 [9.1–35.1]	26.8 [12.6–67.6]	–	0.0119
Diagnosis and Pathology (NCCN Risk Groups)				
High risk, n (%)	143 (57.2%)	41 (82.0%)	184 (61.3%)	–
Low+intermediate risk, n (%)	107 (42.8%)	9 (18.0%)	116 (38.7%)	–
Gleason Grade Group Distribution				
Grade Group 1 (GS 3 + 3 = 6), n (%)	46 (18.4%)	3 (6.0%)	49 (16.3%)	–
Grade Group 2 (GS 3 + 4 = 7), n (%)	48 (19.2%)	8 (16.0%)	56 (18.7%)	–
Grade Group 3 (GS 4 + 3 = 7), n (%)	57 (22.8%)	8 (16.0%)	65 (21.7%)	–
Grade Group 4 (GS 8), n (%)	56 (22.4%)	19 (38.0%)	75 (25.0%)	–
Grade Group 5 (GS 9-10), n (%)	43 (17.2%)	12 (24.0%)	55 (18.3%)	–
Benign/No Cancer, n (%)	0 (0.0%)	0 (0.0%)	0 (0.0%)	–
PI-RADS Score Distribution				
PI-RADS (ordinal), median [IQR]	4.0 [3.0–5.0]	5.0 [4.0–5.0]	–	0.0150
PI-RADS 1-2, n (%)	19 (7.6%)	2 (4.0%)	21 (7.0%)	–
PI-RADS 3, n (%)	69 (27.6%)	6 (12.0%)	75 (25.0%)	–
PI-RADS 4, n (%)	70 (28.0%)	16 (32.0%)	86 (28.7%)	–
PI-RADS 5, n (%)	92 (36.8%)	26 (52.0%)	118 (39.3%)	–
MRI Technical Parameters				
MRI System	GE Healthcare Discovery 750 3.0T			–
Acquisition Parameters	**T2-weighted (Axial)**		**DWI (Axial)**	–
Repetition Time (TR), ms	3000–4000		2000–3000	–
Echo Time (TE), ms	80–120		60–90	–
Field of View (FOV), cm	34×34	34×34	–
Matrix	320×256		128×128	–
b-value, s/mm ^2^	–		1000	–
Slice Thickness/Gap, mm	3.0/1.0		–	–
Endorectal coil usage, n (%)	38 (15.2%)	9 (18.0%)	47 (15.7%)	–

PCa, Prostate Cancer; SD, Standard Deviation; PSA, Prostate Specific Antigen; GS, Gleason Score; PI-RADS, Prostate Imaging-Reporting and Data System; DWI, Diffusion-Weighted Imaging; ADC, Apparent Diffusion Coefficient. The development cohort includes patients scanned between January 2021 and December 2022, while the external test cohort includes patients scanned between January 2023 and May 2023. No benign/no-cancer cases were included; the study task is NCCN risk stratification among pathologically confirmed PCa cases. ^‡^Two-sided Mann–Whitney U tests were used for development vs external comparisons of age, PSA, and PI-RADS (ordinal score). PSA *n* reflects non-missing values. Bold values indicate the best performance per metric. Bold values indicate the best performance per metric.

The higher high-risk prevalence in the independent temporal test cohort reflects a natural case-mix shift over time under a chronological split, rather than a controlled sampling design. Because our goal was to simulate prospective deployment, we did not enforce prevalence matching between cohorts. We therefore report both prevalence-invariant discrimination (ROC-AUC) and additional prevalence-robust/prevalence-aware metrics (e.g., balanced accuracy and predictive values at fixed operating points) to support interpretation under this shifted prevalence.

### 2.3 MRI acquisition and preprocessing

All patients underwent mpMRI on a GE Healthcare Discovery 750 3.0T scanner using a pelvic phased-array coil. All imaging protocols conformed to PI-RADS v2.1 technical specifications (6). Key sequences included axial T2-weighted (T2WI) imaging (TR: 3000–4000 ms; TE: 80–120 ms) and axial diffusion-weighted imaging (DWI) (TR: 2000–3000 ms; TE: 60–90 ms) with a b-value of 1000 s/mm^2^, from which ADC maps were computed. For both sequences, slice thickness was 3.0 mm with a 1.0 mm inter-slice gap. Preprocessing consisted of independent steps for T2-weighted (T2WI) and Apparent Diffusion Coefficient (ADC) volumes to prepare them for the deep learning model.

Both modalities were first resampled to a common voxel spacing of 0.391 × 0.391 × 4.0 mm^3^ using third-order spline interpolation for images and nearest-neighbor interpolation for their corresponding segmentation masks. Prostate gland segmentation masks were generated using an automated commercial contouring software, the PV-iCurve Intelligent Radiotherapy Contouring System (PVmed Tech), which is U-Net-based and leverages few-shot learning with multimodal imaging (19, 20). According to vendor documentation, the system reports an average Dice similarity coefficient (DSC) *>* 0.9 (19). We used these prostate gland masks only for (i) defining the region for z-score intensity normalization and (ii) defining an anatomical region-of-interest for standardized cropping; the masks do not encode tumor location and were generated without using pathology or NCCN risk labels. We did not train or fine-tune the segmentation model on our 300-patient cohort and do not have access to the proprietary training dataset; therefore, we cannot report cohort-specific segmentation accuracy, which we note as a limitation. Intensity values were then normalized using z-score normalization, calculated within the prostate gland region defined by these masks. To account for anatomical variability across patients, we employed a standardized cropping procedure. For each modality, we calculated the physical center and bounding box from its respective mask, determined the maximum physical extent across both modalities, and then extracted fixed-size crops centered at each modality’s respective center. Zero-padding was used to ensure consistent dimensions across all volumes. This process yielded preprocessed volumes ( ${I^{\prime }}_{T2}$, ${I^{\prime }}_{ADC}$) and masks ( ${M^{\prime }}_{T2}$, ${M^{\prime }}_{ADC}$) for each patient.

### 2.4 Patch extraction for model input

**Figure 2:**
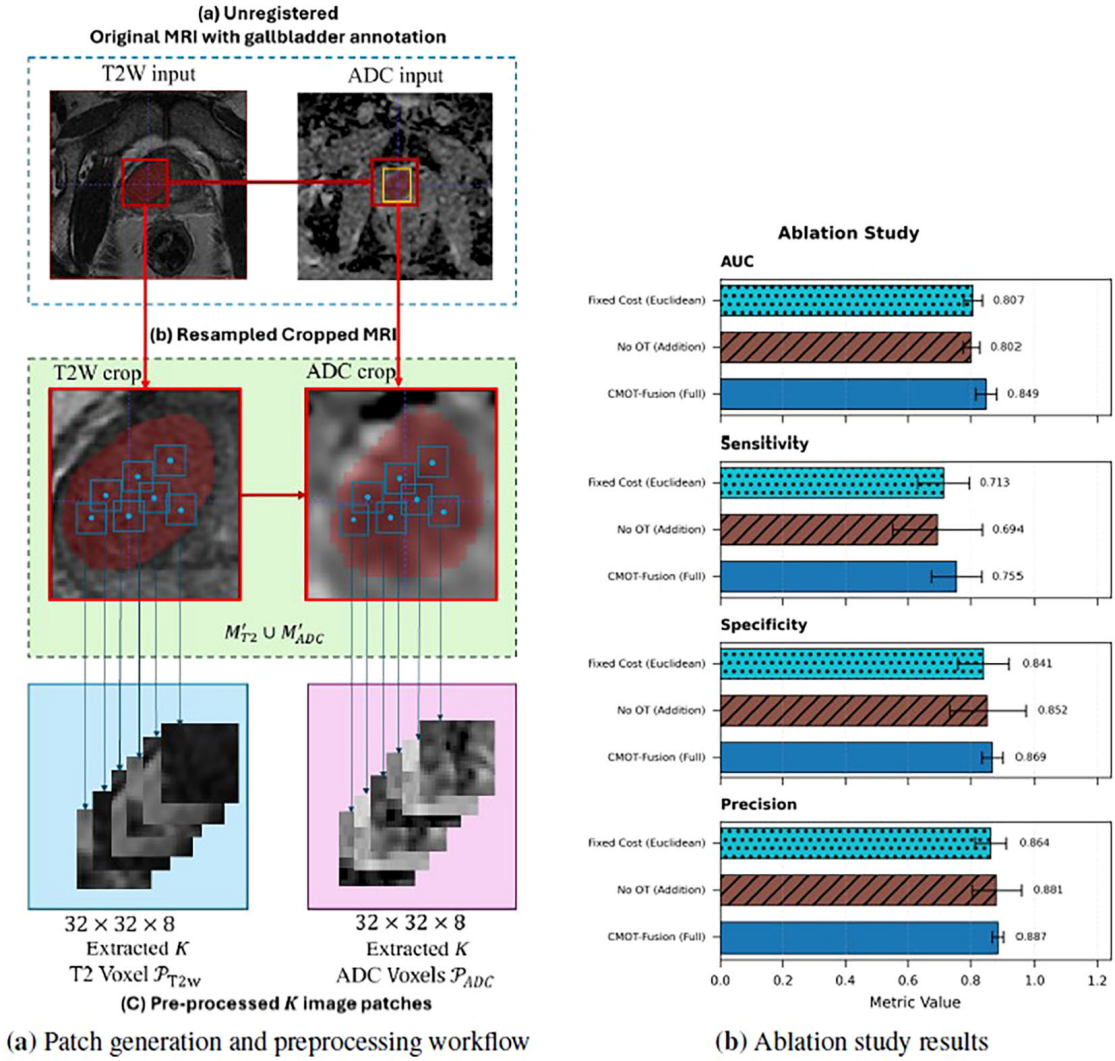
Methodological overview and ablation analysis. **(a)** Patch generation and preprocessing workflow: Original T2WI and ADC input MRIs are resampled to consistent spacing (0.391 × 0.391 × 4.0 mm^3^), aligned using physical centers of respective segmentation masks, and cropped based on maximal bounding box. Patch sampling locations (blue markers) are indicated within the combined mask region ( $M_{T2}^{'}\cup \;M_{ADC}^{'}$). *K* = 32 3D patches (32 × 32 × 8 voxels) are extracted from both modalities (𝒫*_T_*_2_*_WI_*, 𝒫*_ADC_*) as inputs to feature encoders. **(b)** Ablation study results: Visual comparison of CMOT-Fusion (Full) against ‘No OT (Addition)’ and ‘Fixed Cost (Euclidean)’ variants across key performance metrics (AUC, Sensitivity, Specificity, Precision) on the internal dataset, demonstrating the superior performance of the complete framework.

To generate inputs for the model, we adopted a 3D patch-based strategy, visually summarized in **Figure 2a**. For each patient, we sampled K=32 center coordinates from the union of the preprocessed T2WI and ADC mask regions ( $M^{\prime }=M_{T2}^{\prime }\cup M_{ADC}^{\prime }$). For each center coordinate $c_{i}$, we extracted 3D patches with dimensions of 32×32×8 voxels from both the preprocessed T2WI and ADC volumes. This procedure resulted in two sets of patches per patient: ${𝒫}_{T2}={\left\{P_{T2}^{\left(i\right)}\right\}}_{i=1}^{K}$ and ${𝒫}_{ADC}={\left\{P_{ADC}^{\left(i\right)}\right\}}_{i=1}^{K}$. These patch sets served as the direct inputs to the twin feature encoders of the model. During model training, patch centers were selected via random sampling to introduce data augmentation, whereas a deterministic grid-based selection was used during validation and testing to ensure reproducibility. Specifically, for validation/testing we place candidate centers on a regular 3D grid within the bounding box of *M*^′^ and retain those inside *M*^′^; we then select *K* centers by uniform subsampling to provide broad ROI coverage with a fixed, reproducible ordering across runs.

Patient-level aggregation. CMOT-Fusion produces one prediction per patient. After cross-modal fusion, the model aggregates the *K* fused patch embeddings into a single patient representation using uniform average pooling across patches (or masked average pooling when a patch-validity mask is provided), and then applies an MLP classification head to obtain patient-level logits and probabilities. For independent test evaluation, we report a patient-level ensemble score computed as the mean of the predicted probabilities from the five fold-specific models for each patient (as defined in the Statistical Analysis section).

### 2.5 CMOT-fusion framework for cross-modal classification

#### 2.5.1 Framework overview

**Figure 3:**
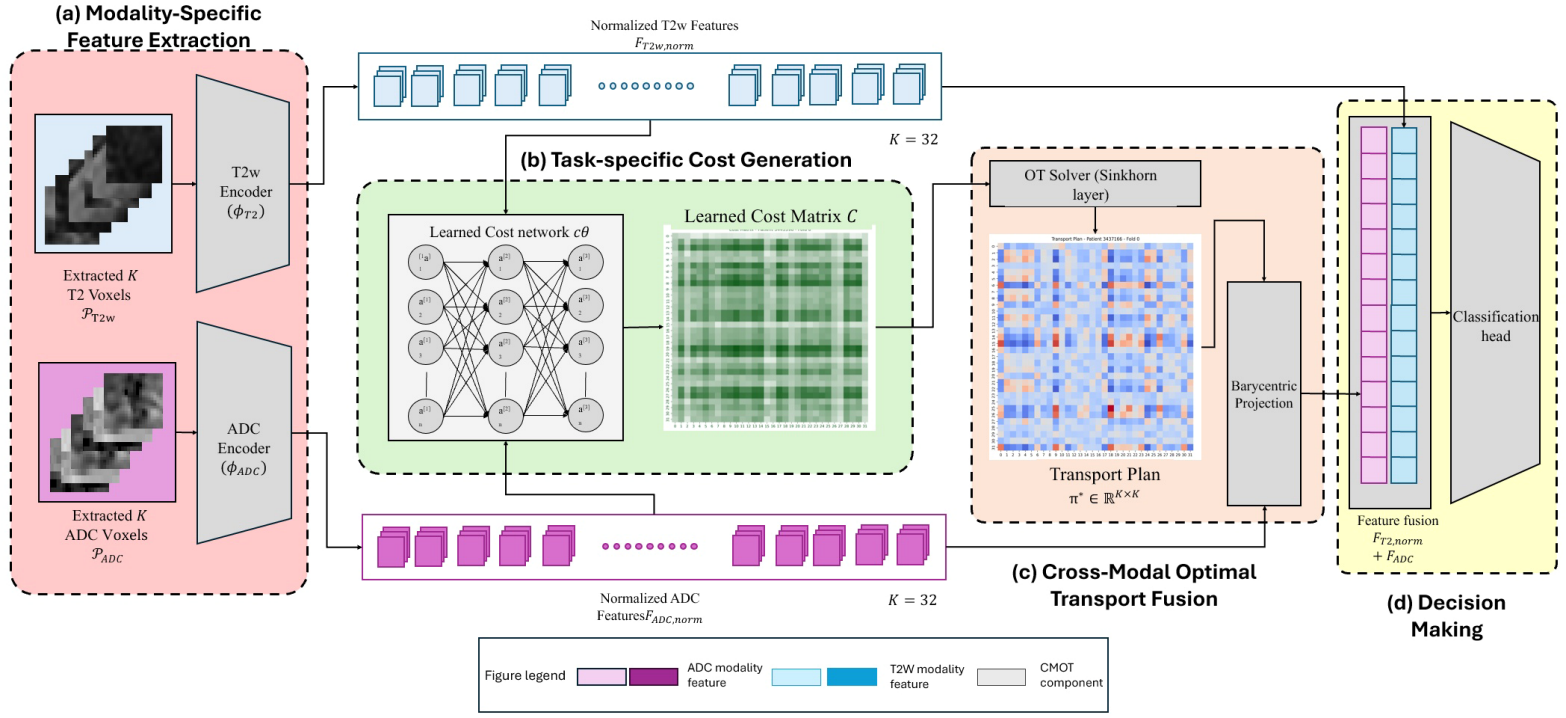
Framework overview of the proposed Cross-Modal Optimal Transport Fusion (CMOT-Fusion) method. The pipeline consists of: **(a)** Modality-Specific Feature Extraction, where *K* patches are extracted from T2WI and ADC volumes and subsequently processed by dedicated encoders (*ϕ_T_*_2_*_WI_*, *ϕ_ADC_*) to yield normalized feature embeddings (*F_T_*_2_*_WI,norm_*, *F_ADC,norm_*); **(b)** Task-specific Cost Generation, wherein a learned cost network (*c_θ_*) estimates a cost matrix *C* defining correspondence costs between the modalityspecific features; **(c)** Cross-Modal Optimal Transport Fusion, which employs an Optimal Transport (OT) solver, implemented with a Sinkhorn layer, to compute the transport plan *π*^∗^, followed by barycentric projection to align ADC features with the T2WI feature space; and **(d)** Decision Making, where transported ADC features (*F_ADC_*) are fused with T2WI features (*F_T_*_2_*_WI,norm_*) and processed by a classification head for final prediction. The figure legend details the color-coding for ADC modality features, T2WI modality features, and CMOT components. This approach explicitly models cross-modal correspondences through a learned, task-specific cost function.

The proposed Cross-Modal Optimal Transport Fusion (CMOT-Fusion) framework aims to effectively fuse information from T2WI and ADC MRI for prostate cancer classification by explicitly modeling the correspondence between localized regions using learned costs. **Figure 3** provides a detailed overview of the architecture and data flow through our proposed method. The framework operates on pairs of 3D patches extracted from the unregistered T2WI and ADC volumes. Independent, twin 3D ResNet-based encoders ( $\phi_{T2}$, $\phi_{ADC}$) (21), utilizing basic ResNet blocks (specifically, using basic blocks with a layer configuration of [2, 2, 2, 2]) (22), first process the patches from each modality separately to generate feature embeddings, denoted as $F_{T2}={\left\{f_{T2}^{\left(i\right)}\right\}}_{i=1}^{K}$ and $F_{ADC}={\left\{f_{ADC}^{\left(j\right)}\right\}}_{j=1}^{K}$, where *K* is the number of patches per volume. These initial features undergo normalization (Batch Normalization followed by Layer Normalization) (23, 24), resulting in $F_{T2,norm}$ and $F_{ADC,norm}$, to enhance stability for subsequent computations, particularly the cost calculation.

A dedicated Learned Cost Network ( $c_{\theta }$), implemented as a Multi-Layer Perceptron (MLP) with a configurable depth, takes pairs of normalized feature vectors (one from $F_{T2,norm}$, one from $F_{ADC,norm}$) as input and estimates a scalartransport cost between them. This process yields a K×K cost matrix C, where $C_{ij}$ represents the learned cost of matching the i-th T2WI patch feature with the j-th ADC patch feature. The detailed formulation of this cost computation is provided in Section 2.5.3.

The entropic regularized Optimal Transport (OT) problem (25) is then formulated to find the optimal probabilistic mapping $\pi^{*}$ between the distributions of T2WI and ADC patch features, balancing the total transport cost against an entropy term. This optimization problem (detailed in Equation 3) is efficiently solved using a numerically stabilized Sinkhorn algorithm with a fixed number of iterations to obtain the *K* × *K* transport plan $\pi^{*}$ (26).

Fusion is achieved via barycentric projection. The normalized ADC features (*F_ADC,norm_*) are projected onto the T2WI feature space using the computed transport plan as described in Equation 7. These transported features are then combined with the normalized T2WI features (*F_T_*_2_*_,norm_*) through element-wise addition as formulated in Equation 8. The complete mathematical details of this fusion process are elaborated in Section 2.5.5.

To enhance numerical stability and improve generalization, layer normalization (24) is applied to the fused features, producing the normalized representation $F_{fused,norm}\in {\mathbb{R}}^{B\times K\times d_{e}}$.

The resulting normalized fused representation integrates complementary information from both modalities in a principled manner, guided by the learned cross-modal correspondences. If an optional validity mask $M\in {\left\{0,1\right\}}^{B\times K}$ is provided, it is applied to the fused features before aggregation, though in our standard implementation all patches are considered valid by default.

#### 2.5.2 Twin feature encoders ( $\phi_{T2},\phi_{ADC}$)

The framework employs two independent yet architecturally identical 3D convolutional neural network encoders, denoted as $\phi_{T2}$ and $\phi_{ADC}$, for generating initial feature representations from T2WI and ADC modalities, respectively. Each encoder processes the corresponding set of K input patches, represented as ${𝒫}_{\text{modality}}={\left\{P_{\text{modality}}^{\left(i\right)}\in {\mathbb{R}}^{1\times D\times H\times W}\right\}}_{i=1}^{K}$, where modality ∈{T2WI,ADC}. The encoders are based on the ResNet architecture (22), adapted for 3D inputs with spatial dimensions. The implementation utilizes basic ResNet blocks with a configuration comprising four stages of convolutional layers. The channel dimensions in these stages follow a standard progression pattern scaled by a factor *λ* to accommodate the computational constraints of 3D processing while maintaining representational capacity.

Formally, each encoder $\phi_{\text{modality}}$ performs the following mapping:

(1) $$F_{\text{modality}}={\left\{f_{\text{modality}}^{\left(i\right)}=\phi_{\text{modality}}\left(P_{\text{modality}}^{\left(i\right)}\right)\in {\mathbb{R}}^{d_{e}}\right\}}_{i=1}^{K}$$

**Equation 1** defines the modality-specific encoder mapping from each 3D patch to a \(d_e\)-dimensional embedding.

where $f_{\text{modality}}^{\left(i\right)}$ represents the feature embedding of the *i*-th patch, and *d_e_* denotes the embedding dimension. These extracted feature representations capture modality-specific information from each localized patch and serve as inputs for the subsequent normalization and cost computation stages described in the following subsections.

#### 2.5.3 Learned cost network (*c_θ_*)

A key innovation in CMOT-Fusion is the Learned Cost Network (*c_θ_*), which dynamically estimates the correspondence costs between modalities, replacing traditional fixed metrics such as Euclidean or cosine distance. This network enables the model to learn data-driven, task-specific correspondence costs that adapt to the intrinsic characteristics of T2WI and ADC modality representations.

The cost network *c_θ_* is implemented as a Multi-Layer Perceptron (MLP) with depth, 2 hidden layers, each with a hidden dimension of 512 units. The network architecture can be formally defined as:

(2) $$c_{\theta }\left(f_{T2}^{\left(i\right)},f_{ADC}^{\left(j\right)}\right)={\text{MLP}}_{\theta }\left(\left[f_{T2}^{\left(i\right)};f_{ADC}^{\left(j\right)}\right]\right)$$

**Equation 2** defines the learned cost network used to compute pairwise cross-modal transport costs.

where [·;·] denotes concatenation, resulting in an input dimension of $2d_{e}=512$ (twice the embedding dimension $d_{e}=256$). Each hidden layer applies a linear transformation followed by ReLU activation (non-inplace) and dropout regularization with probability p=0.3.

The cost network $c_{\theta }$ processes all K×K pairs of normalized feature vectors to produce the cost matrix $C\in {\mathbb{R}}^{K\times K}$, where each element $C_{ij}$ represents the transport cost between the i-th T2WI patch and the j-th ADC patch. To ensure numerical stability during the subsequent Sinkhorn iterations, these costs are clipped to the range [−5.0,5.0] before being passed to the Optimal Transport solver with entropic regularization parameter α=0.5. Ablation studies in Section 3.2 demonstrate the significant performance advantage of this learned approach compared to fixed metrics.

#### 2.5.4 Optimal transport with Sinkhorn algorithm

Given the learned cost matrix $C\in {\mathbb{R}}^{K\times K}$, the optimal transport plan $\pi^{*}\in {\mathbb{R}}^{K\times K}$ between the T2WI and ADC patch feature distributions is computed by solving the entropic regularized Optimal Transport (OT) problem (25) as follows:

(3) $$\pi^{*}=\underset{\pi \in U\left(r,c\right)}{\arg\min}\langle \pi ,C\rangle -\alpha H\left(\pi \right)$$

where the inner product $\langle \pi ,C\rangle =\sum_{i,j}\pi_{ij}C_{ij}$ computes the total transport cost, $H\left(\pi \right)=-\sum_{i,j}\pi_{ij}\left(\text{log }\pi_{ij}-1\right)$ is the entropy of the transport plan, and α>0 is the regularization parameter controlling the trade-off between cost minimization and entropy maximization. The transport polytope U(r,c) represents the set of all non-negative matrices with fixed marginals:

(4) $$U\left(r,c\right)=\{\pi \in {\mathbb{R}}_{+}^{K\times K}|\pi 1_{K}=r,\pi^{T}1_{K}=c\}$$

**Equation 4** defines the admissible transport polytope under fixed marginals.

where $r,c\in {\text{Σ}}_{K}$ are probability vectors (typically uniform, $r_{i}=c_{j}=1/K$) and $1_{K}$ is a vector of ones.

To solve this regularized OT problem efficiently, we employ the Sinkhorn algorithm (26, 27), summarized in Algorithm 1. This algorithm provides several key advantages for our medical imaging application: (1) computational efficiency compared to linear programming approaches, (2) differentiability enabling end-to-end training, and (3) controllable smoothness through the regularization parameter *α*.

The Sinkhorn algorithm reformulates the OT problem in terms of dual variables u and v. For numerical stability, we implement the algorithm in the log domain with the following iterative updates:

(5) $$\begin{array}{ll} f^{\left(t+1\right)} & =\text{log }r-\text{LSE}\left(g^{\left(t\right)}-C/\alpha \right)\\ g^{\left(t+1\right)} & =\text{log }c-\text{LSE}\left(f^{\left(t+1\right)}-C^{⊤}/\alpha \right) \end{array}$$

**Equation 5** gives the log-domain Sinkhorn update equations used to solve the entropic OT problem.

where f=log u, g=log v are the log-domain variables, LSE is the log-sum-exp operation (applied row/column-wise), and $r=c=\frac{1}{K}1_{K}$ are uniform marginals. After convergence, the transport plan is recovered with:

(6) $$\pi_{ij}^{*}=\text{exp }(f_{i}+g_{j}-C_{ij}/\alpha )$$

The dual variables u and v correspond to Lagrange multipliers enforcing the marginal constraints, and the algorithm iteratively updates them until convergence or a fixed iteration limit. The final transport plan is then computed as $\pi^{*}$ = diag( u)*K*diag( v).

The Sinkhorn algorithm is implemented within a custom differentiable layer, termed ‘SinkhornLayer’, integrated into the network architecture. This layer addresses practical numerical challenges inherent in deep learning frameworks and provides the interface between the learned cost matrix and the subsequent fusion operations. Several key stabilization techniques are employed:

1. *Log-domain computation*: Operations involving the kernel matrix are performed in the log-domain to mitigate numerical underflow and overflow.
2. *Value stabilization*: A small constant $\in =10^{-6}$ is added during division operations, including marginal computations and final normalization, to prevent division by zero.
3. *Cost clipping*: Input costs are clipped to [−5.0,5.0] before kernel computation to maintain numerical stability, aligning with the learned cost network’s output range.

Furthermore, during iterative updates, the log-domain dual variables ( f and g) are clamped within [−20,20] to prevent extreme values and ensure stable gradient propagation. When reconstructing the transport plan $\pi^{*}$ via Equation 6, the exponent term $\left(f_{i}+g_{j}-C_{ij}/\alpha \right)$ is clamped within [−30,30] before exponentiation to avoid numerical overflow or underflow. Finally, an additional normalization step is applied to the resulting plan $\pi^{*}$ to enforce strict doubly-stochastic properties and compensate for potential minor numerical inaccuracies from the iterative process.

Through comprehensive hyperparameter tuning experiments (detailed in Section 3.3), we determined optimal values for the entropic regularization strength *α* = 0.5 and iteration count *T* = 30. The regularization parameter *α* controls the smoothness of the resulting transport plan—smaller values produce sparse, deterministic mappings while larger values yield more uniform, diffused correspondence. Our chosen value balances specificity in patch matching against robustness to noise. The iteration count *T* = 30 was selected as the minimal value ensuring consistent convergence across our dataset while maintaining computational efficiency.

The algorithm takes the cost matrix *C* as input and produces the optimal transport plan $\pi^{*}$ as output, with uniform marginals ( ${\text{r}}_{i}={\text{c}}_{j}=1/K$) enforcing a balanced correspondence between all patches. In our implementation, we set all patches as valid by using a zero threshold for mask overlap, effectively treating all extracted patches equally in the transport plan computation. The resulting plan *π*^∗^ encodes the learned probabilistic correspondence between patches across modalities and serves as the foundation for the subsequent feature fusion via barycentric projection.

#### 2.5.5 Fusion via barycentric projection

Once the optimal transport plan *π*^∗^ ∈ ℝ*^K^*^×^*^K^* is computed, it serves as a probabilistic mapping to guide the fusion of information between modalities. Barycentric projection is employed to transport the normalized ADC patch features (*F_ADC,norm_*) into the T2WI feature space, effectively aligning the features based on the learned correspondence.

Algorithm 1

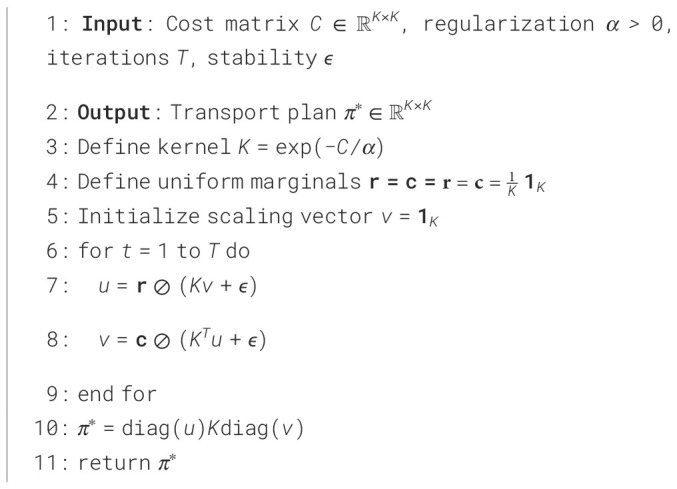

Mathematically, this projection is formulated as a matrix multiplication:

(7) $${\hat{F}}_{ADC}=\pi^{*}F_{ADC,norm}$$

**Algorithm 1** summarizes the Sinkhorn iterations used to compute the entropic optimal transport plan.

where ${\hat{F}}_{ADC}\in {\mathbb{R}}^{B\times K\times d_{e}}$ represents the transported ADC features in the T2WI feature space, with B denoting the batch size. Each element ${\hat{f}}_{ADC}^{\left(i\right)}$ in ${\hat{F}}_{ADC}$ is a weighted combination of the original normalized ADC features, with weights specified by the i-th row of the transport plan $\pi^{*}$.

The transported ADC features are then combined with the normalized T2WI features through element wise addition:

(8) $$F_{fused}=F_{T2,norm}+{\hat{F}}_{ADC}$$

To enhance numerical stability and improve generalization, layer normalization is applied to the fused features, producing the normalized representation $F_{fused,norm}\in {\mathbb{R}}^{B\times K\times d_{e}}$.

The resulting normalized fused representation integrates complementary information from both modalities in a principled manner, guided by the learned cross-modal correspondences. If an optional validity mask $M\in {\left\{0,1\right\}}^{B\times K}$ is provided, it is applied to the fused features before aggregation, though in our standard implementation all patches are considered valid by default.

### 2.6 Model development and evaluation strategy

We evaluated the proposed CMOT-Fusion framework against several established methods for mpMRI-based prostate cancer classification. To ensure fair comparisons, all models utilized identical data splits, ROI definitions, and consistent backbone architectures where applicable. The following methods were compared:

Single-Modality Baselines. Standard 3D ResNet (21) and Vision Transformer (ViT) (28) architectures were trained independently on T2WI or ADC patches. The ResNet utilized an identical architecture to the twin encoders employed in CMOT-Fusion (basic blocks, [2,2,2,2] configuration). The ViT employed a patch size of 8 × 8 × 2 and standard transformer encoder layers.

Conventional Fusion Methods. This category included three approaches. *Late Fusion*: Simple voting ensembles combining the probabilistic outputs from independently trained single-modality ResNet and ViT models (one ensemble for ResNet pair, one for ViT pair). *Intermediate Fusion (Concatenation)*: Features extracted by parallel ResNet/ViT backbones for T2WI and ADC were concatenated before being fed into a shared classification head. *Attention-Based Fusion*: Cross-attention mechanisms were integrated between features from parallel ResNet/ViT backbones to weigh the contribution of one modality based on the other before fusion and classification.

CMOT-Fusion Variants. Variants of our proposed method were analyzed to assess the contribution of individual components, as detailed in Section 3.2.

All compared methods utilized the same patch extraction, preprocessing, data augmentation (where applicable), optimizer settings, learning rate schedule, and training duration as CMOT-Fusion, unless architecturally precluded (e.g., single-modality methods only use augmentations for their respective input).

### 2.7 Statistical analysis

Model performance was primarily evaluated using the Area Under the Receiver Operating Characteristic Curve (AUC) to assess overall diagnostic capability. Secondary metrics included Accuracy, F1-Score, Sensitivity (Recall), and Specificity. To provide additional context under prevalence shift, we report balanced accuracy, defined as $\frac{1}{2}$ (Sensitivity + Specificity), and the area under the precision-recall curve (PR-AUC; average precision) on the independent test set. For these threshold-dependent metrics, the optimal operating point for each model was determined from the validation set of its corresponding cross-validation fold using Youden’s J index, defined as *J*, Sensitivity + Specificity − 1.

Performance on the development cohort was summarized as the mean and standard deviation of each metric across the 5 cross-validation folds. For the independent temporal test cohort, we report performance in two complementary ways: (1) *ensemble*: for each test patient, we obtain five predicted probabilities (one from each fold-specific model) and compute the ensemble probability as their mean; we then compute the test AUC once from these ensemble probabilities; and (2) *mean fold*: we compute the test AUC separately for each of the five fold-specific models on the same held-out test cohort, and report the mean ± standard deviation across these five AUC values. For the ensemble AUC on the test set, 95% confidence intervals (CIs) were estimated using 2000 bootstrap resamples.

Because fold-wise hypothesis tests are underpowered with only five cross-validation splits (yielding discrete *p*-values), we perform the primary comparative inference at the patient level using pooled out-of-fold (OOF) predictions across the development cohort (each patient is evaluated once by a model that was not trained on that patient). For each baseline comparison, we report the paired effect size as ΔAUC (CMOT–baseline) with a 95% confidence interval estimated by paired bootstrap resampling of patients (2000 resamples). Two-sided *p*-values are obtained using a paired permutation test that randomly swaps paired model scores within each patient under the null of exchangeability (10,000 permutations). To account for multiple baseline comparisons, we apply Holm correction to control the family-wise error rate. Fold-wise AUC summaries are still reported descriptively to show consistency across splits, but fold-wise hypothesis tests are treated as sensitivity analyses.

The definitions for the secondary metrics are as follows, where TP, TN, FP, and FN represent true positives, true negatives, false positives, and false negatives, respectively:

(9) $$\text{Accuracy}=\frac{TP+TN}{TP+TN+FP+FN}$$

(10) $$\text{Sensitivity }\left(\text{Recall}\right)=\frac{TP}{TP+FN}$$

(11) $$\text{Precision}=\frac{TP}{TP+FP}$$

(12) $$\text{Specificity}=\frac{TN}{TN+FP}$$

(13) $$\text{Balanced Accuracy}=\frac{1}{2}\left(\frac{TP}{TP+FN}+\frac{TN}{TN+FP}\right)$$

(14) $$\text{F}1–\text{Score}=2\times \frac{\text{Precision}\times \text{Recall}}{\text{Precision}+\text{Recall}}$$

**Equations 9**–**14** define the evaluation metrics used in this study (accuracy, sensitivity/recall, precision, specificity, balanced accuracy, and F1-score).

### 2.8 Implementation details

All models were implemented in Python using PyTorch (with MONAI for 3D ResNet components) and trained with mixed-precision acceleration (automatic mixed precision using torch.cuda.amp autocast and gradient scaling). We trained using AdamW with an initial learning rate of 1 × 10^−4^ and weight decay 5 × 10^−4^. The learning rate was scheduled with ReduceLROnPlateau (mode=max, factor=0.3, patience=6, min learning rate 1×10^−6^). Training was run for up to 50 epochs with early stopping patience of 10 epochs based on validation AUC. We used a batch size of 8 for training and 8 for validation, with 4 data-loader workers and pinned memory.

Experiments were run on a workstation equipped with an NVIDIA RTX A6000 GPU. For completeness, the CPU environment was an Intel Xeon-class processor with 128 GB system RAM. In our main configuration (*K* = 32 patches per patient and 30 Sinkhorn iterations), end-to-end training required approximately 2.5 hours per fold (about 12.5 hours total for 5-fold cross-validation, depending on early stopping), and patient-level inference required approximately 0.8 seconds per patient (excluding offline preprocessing). To characterize the computational overhead of the optimal-transport (OT) fusion itself, we additionally microbenchmarked the OT block (SinkhornLayer + barycentric projection) on the same GPU using batch size 1 and embedding dimension 256. The OT block required approximately 6.6–8.6 ms per forward pass for *K* = 8–64 and exhibited a peak GPU footprint of approximately 8.3 MiB allocated (22 MiB reserved) under this isolated benchmark. Because the OT computation forms and operates on a *K* × *K* transport plan, its time and memory complexity scale approximately as *O*(*K*^2^) with the number of patches.

## 3 Results

### 3.1 Diagnostic performance of CMOT-fusion

We evaluated CMOT-Fusion against multiple established approaches, including single-modality methods and conventional fusion strategies (late fusion, intermediate concatenation, and attention-based mechanisms).

**Figure 4:**
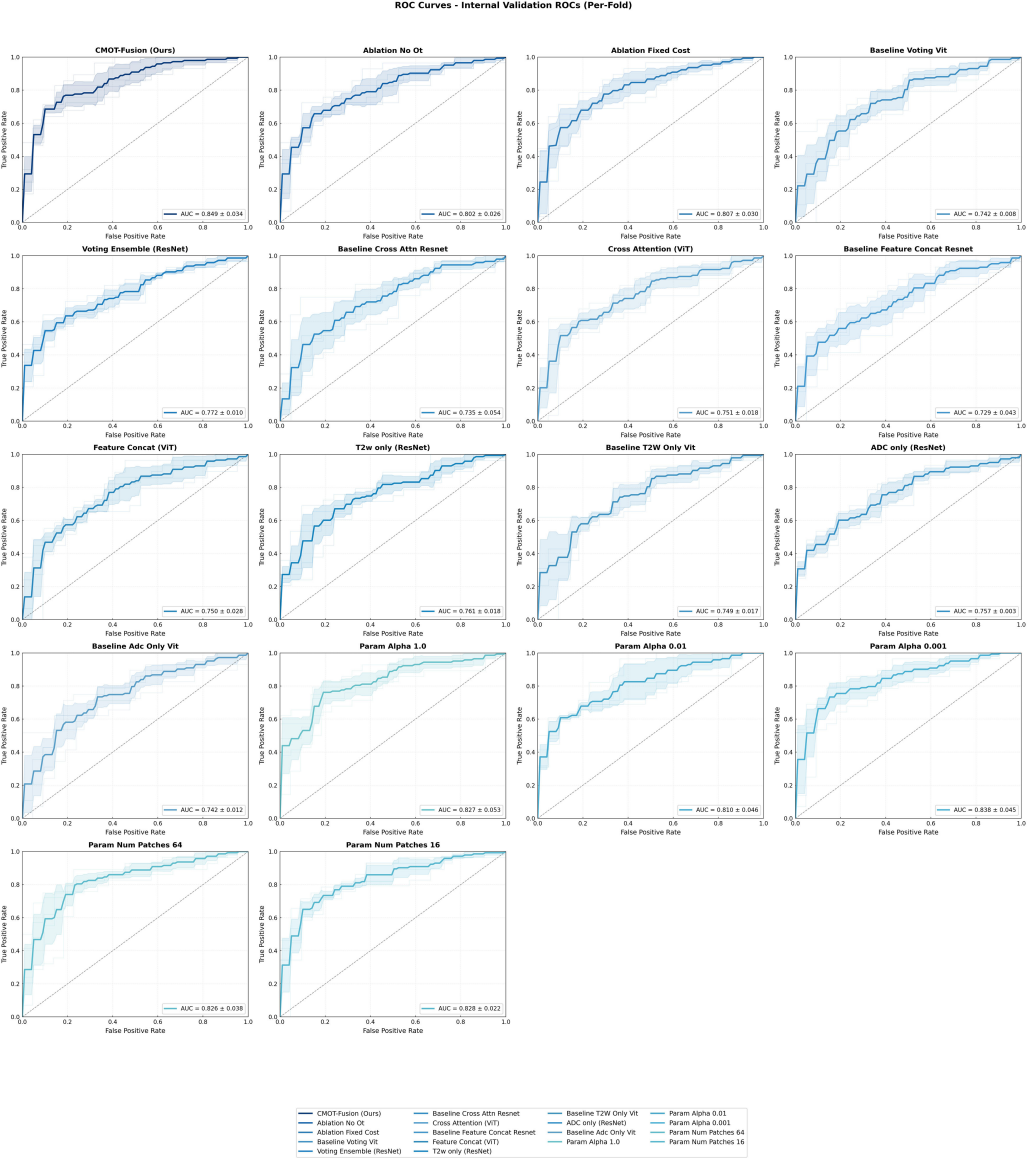
Per-fold ROC curves on the internal cross-validation dataset. This figure illustrates the variability and performance of the CMOT-Fusion model across each of the 5 cross-validation folds on the internal dataset.

**Figure 5:**
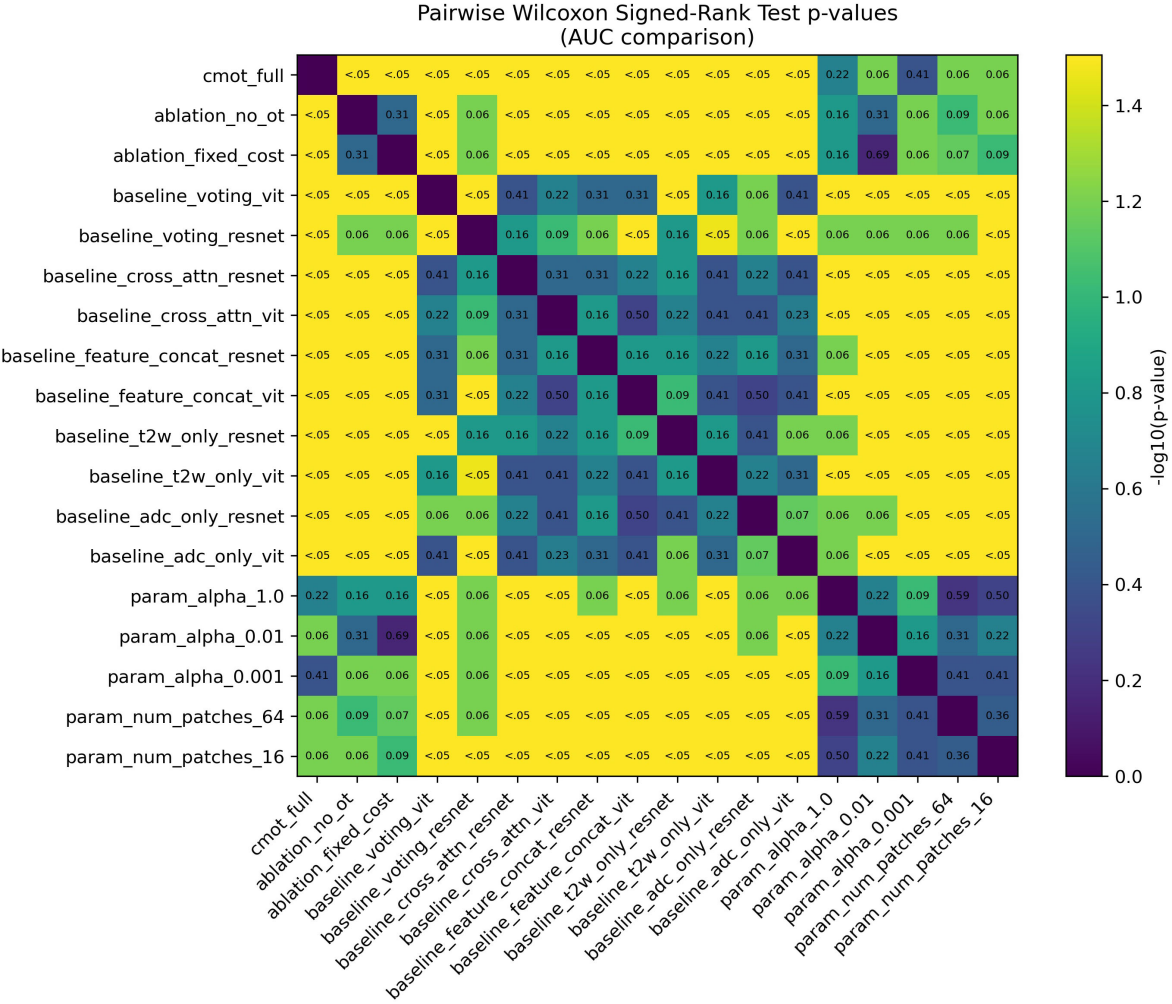
Pairwise statistical comparison p-values for AUC comparison. This heatmap visualizes fold-wise paired comparisons of AUC across the 5 cross-validation folds and is retained as a sensitivity/illustrative summary because with only five folds, fold-level *p*-values are discrete and underpowered. Accordingly, primary comparative inference is based on patient-level pooled out-of-fold predictions using two-sided paired permutation tests with Holm correction and paired bootstrap confidence intervals (Statistical Analysis). The color intensity corresponds to the -log10(p-value), where brighter colors (e.g., yellow) indicate smaller p-values. Numerical p-values are shown within each cell; ‘*p<* 0.05’ highlights comparisons below the nominal threshold.

**Figure 6:**
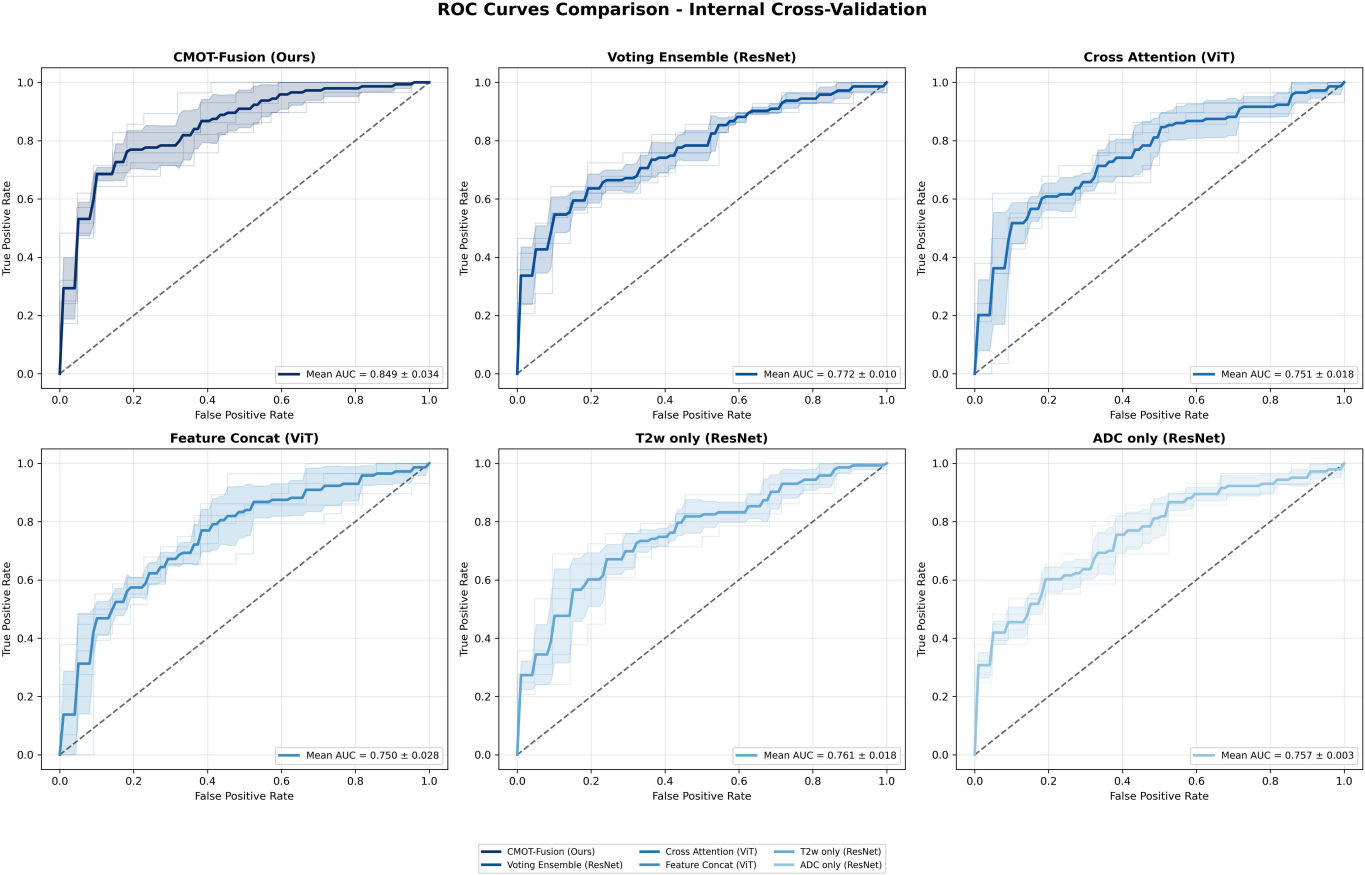
ROC Curves Comparison - Internal Cross-Validation. Comparison of ROC curves for CMOT- Fusion (Ours) against representative baseline models, including top-performing single-modality (T2WI only ResNet, ADC only ResNet) and alternative fusion strategies (Voting Ensemble ResNet, Cross AttentionViT, Feature Concatenation ViT) on the internal cross-validation dataset. Mean AUC values and standard deviations are shown for each method, highlighting CMOT-Fusion’s superior discriminative capability.

Quantitative Performance Analysis. **Table 3** presents comprehensive performance metrics for all evaluated methods. CMOT-Fusion achieved superior performance on the internal cross-validation cohort with a mean AUC of 0.849 ± 0.034 (per-fold performance detailed in **Figure 4**). This represented a clinically meaningful improvement over all comparative methods, including the next best performing model, the Voting Ensemble (ResNet), which had a mean AUC of 0.772 ± 0.010. Using pooled out-of-fold predictions across the development cohort (n=250), CMOT-Fusion achieved an OOF AUC of 0.822. Across the 10 baseline methods, the paired effect size ranged from ΔAUC = 0.078 to 0.153 (CMOT–baseline), with paired bootstrap 95% CIs that remained above 0 for all comparisons (e.g., vs Voting Ensemble (ResNet), ΔAUC = 0.078; 95% CI: 0.010–0.151). Two-sided paired permutation tests yielded unadjusted *p*-values from 0.0002 to 0.046; after Holm correction for multiple comparisons, the most conservative adjusted *p*-values were near the 0.05 threshold, reinforcing our emphasis on effect sizes and confidence intervals rather than fold-wise *p*-values from only five splits. The pairwise P-value heatmap across the five cross-validation folds is shown in **Figure 5**. **Figure 6** visually illustrates these results, where the ROC curve for CMOT-Fusion is consistently positioned above the others, indicating superior performance across all operating thresholds.

**Table 3:** Classification performance comparison on internal and external datasets.

Method	Dataset	Classification metrics			
		AUC↑	Accuracy↑	Sensitivity↑	Specificity↑
CMOT-Fusion (Ours)	Internal External^†^	0.849 ± 0.034 **0.824/0.793 ±** **0.048**	**0.804 ± 0.032** 0.716 ± 0.097	0.755 ± 0.081 0.683 ± 0.132	**0.869 ± 0.034** 0.867 ± 0.083
Voting Ensemble (ResNet)	Internal External^†^	0.772 ± 0.010 0.762/0.736 ± 0.018	0.736 ± 0.023 0.580 ± 0.044	0.665 ± 0.102 0.488 ± 0.053	0.833 ± 0.109 **1.000 ± 0.000**
Voting Ensemble (ViT)	Internal External^†^	0.742 ± 0.008 0.680/0.689 ± 0.016	0.740 ± 0.000 0.700 ± 0.033	0.756 ± 0.123 0.698 ± 0.053	0.721 ± 0.164 0.711 ± 0.054
Cross-Attention (ResNet)	Internal External^†^	0.735 ± 0.054 0.664/0.692 ± 0.052	0.712 ± 0.070 0.608 ± 0.079	0.658 ± 0.117 0.541 ± 0.123	0.784 ± 0.102 0.911 ± 0.130
Cross-Attention (ViT)	Internal External^†^	0.751 ± 0.018 0.732/0.709 ± 0.016	0.720 ± 0.025 0.628 ± 0.063	0.595 ± 0.051 0.585 ± 0.101	0.888 ± 0.046 0.822 ± 0.113
Feature Concatenation (ResNet)	Internal External^†^	0.729 ± 0.043 0.732/0.718 ± 0.023	0.708 ± 0.027 0.656 ± 0.117	0.650 ± 0.163 0.615 ± 0.175	0.783 ± 0.211 0.844 ± 0.151
Feature Concatenation (ViT)	Internal External^†^	0.750 ± 0.028 0.740/0.743 ± 0.020	0.732 ± 0.037 **0.736 ± 0.079**	**0.778 ± 0.138** **0.746 ± 0.119**	0.674 ± 0.106 0.689 ± 0.109
T2WI only (ResNet)	Internal External^†^	0.761 ± 0.018 0.751/0.733 ± 0.006	0.740 ± 0.022 0.584 ± 0.073	0.642 ± 0.080 0.502 ± 0.108	0.868 ± 0.083 0.956 ± 0.089
T2WI only (ViT)	Internal External^†^	0.749 ± 0.017 0.696/0.698 ± 0.005	0.744 ± 0.023 0.684 ± 0.064	0.757 ± 0.120 0.678 ± 0.097	0.732 ± 0.176 0.711 ± 0.089
ADC only (ResNet)	Internal External^†^	0.757 ± 0.003 0.748/0.730 ± 0.027	0.720 ± 0.022 0.620 ± 0.075	0.679 ± 0.122 0.537 ± 0.091	0.776 ± 0.116 **1.000 ± 0.000**
ADC only (ViT)	Internal External^†^	0.742 ± 0.012 0.669/0.675 ± 0.022	0.724 ± 0.027 0.664 ± 0.032	0.636 ± 0.105 0.644 ± 0.048	0.841 ± 0.107 0.756 ± 0.044

Internal dataset: Metrics reported as mean ± standard deviation across 5-fold cross-validation. External dataset AUC: Ensemble AUC/Mean AUC across 5 models. Other external metrics: Mean ± Std using Youden’s J optimal threshold. Bold values indicate the best performance per metric. ↑ Higher is better. Formal statistical comparisons are described in the Statistical Analysis section, emphasizing two-sided patient-level paired tests on pooled out-of-fold predictions (with Holm correction) and effect-size confidence intervals.

**Figure 7:**
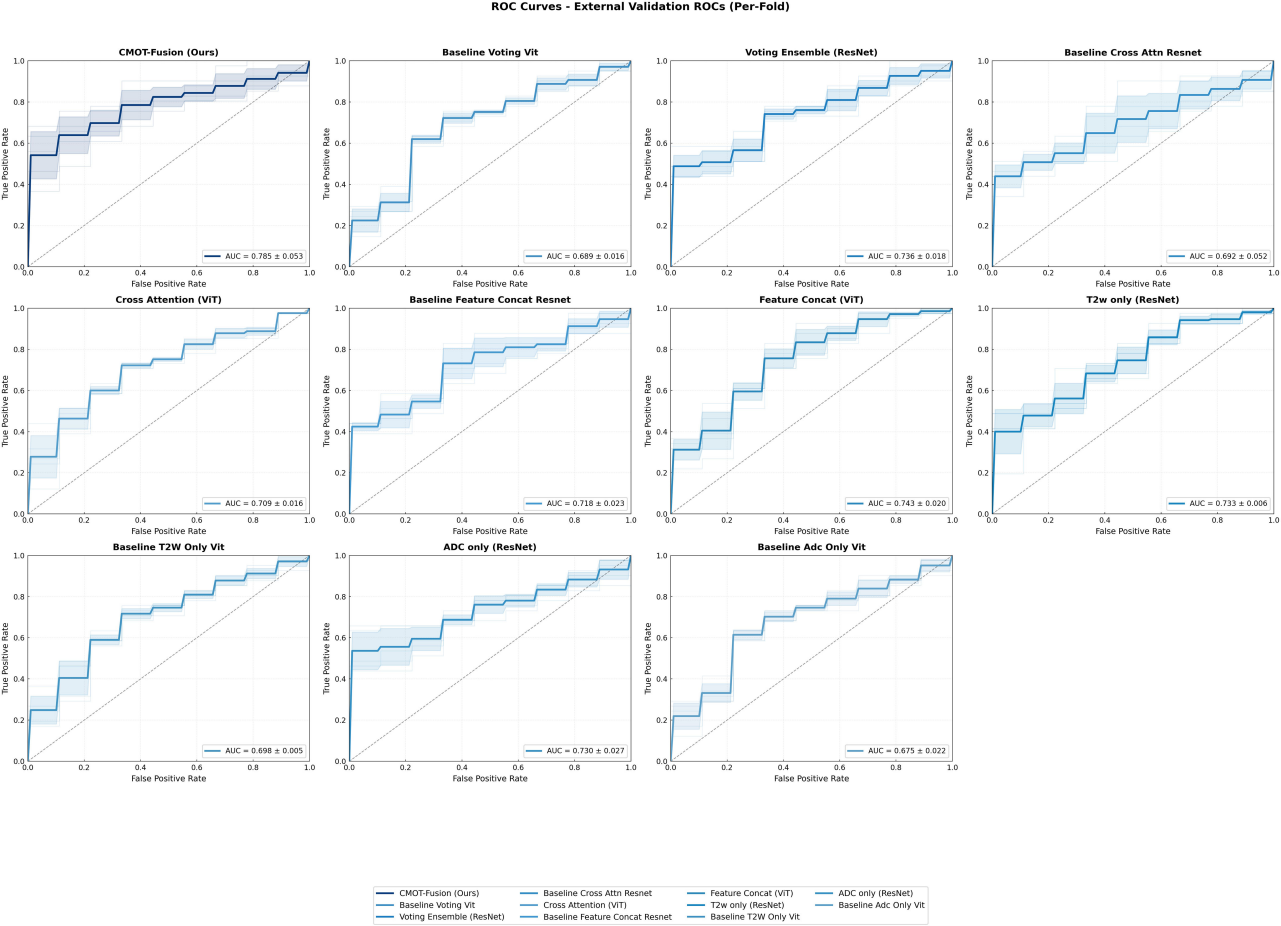
Per-fold ROC curves on the external validation dataset. This figure shows the performance of each of the 5 models (trained on different internal CV folds) when evaluated on the independent external test set.

On the independent temporal test set, CMOT-Fusion maintained its performance advantage with the highest ensemble AUC (0.824; 95% CI: 0.694–0.930) and mean fold AUC (0.793 ± 0.048), as illustrated by the per-fold model performance in **Figure 7**. Here, the ensemble AUC is computed from patient-level ensemble probabilities (mean of the five fold-model probabilities per patient), whereas the mean fold AUC is the mean ± standard deviation of the AUC obtained when each fold-specific model is evaluated individually on the same held-out test cohort. While some comparison methods achieved higher scores on individual secondary metrics, none matched CMOT-Fusion’s balanced and superior overall diagnostic performance. Several baseline methods yielded specificity estimates of 1.000 (or near 1.000) on this small, prevalence-skewed test cohort. Because specificity is computed from only 9 low/intermediate-risk patients, these estimates are inherently coarse (in increments of 1/9) and sensitive to single-case fluctuations; for example, one false positive would reduce specificity from 1.000 to 0.889. Moreover, secondary metrics such as accuracy, sensitivity, and specificity depend on the selected decision threshold (e.g., a Youden’s *J* operating point), and threshold-dependent estimates can be unstable in low-*n* evaluations with skewed class proportions. Accordingly, we emphasize threshold-free discrimination (ROC-AUC) and complement it with prevalence-aware summaries and explicit confusion matrices to contextualize operating-point behavior.

Because the independent temporal test cohort has a higher high-risk prevalence (82.0%), we additionally report prevalence-robust and prevalence-aware summaries to contextualize threshold-based performance, including PR-AUC (average precision) and operating-point confusion matrices. On the test cohort, CMOT-Fusion achieved a PR-AUC of 0.961 (95% CI: 0.917–0.990). Using a fixed probability threshold of 0.5, CMOT-Fusion achieved Sensitivity=0.707, Specificity=0.667, and Balanced Accuracy=0.687 (TP/FP/TN/FN = 29/3/6/12; PPV = 0.906; NPV = 0.333). Using the Youden threshold computed on this cohort as a transparent reference (0.617), CMOT-Fusion achieved Sensitivity=0.659, Specificity=1.000, and Balanced Accuracy=0.829 (TP/FP/TN/FN = 27/0/9/14; PPV = 1.000; NPV = 0.391). We emphasize that while ROC-AUC is prevalence-invariant, PR-AUC, predictive values (PPV/NPV), and other threshold-dependent metrics vary with prevalence and should be interpreted in the context of the cohort case-mix.

Clinical reference using PI-RADS. To provide clinical context, we evaluated PI-RADS v2.1 scores on the same independent test cohort, treating PI-RADS as an ordinal radiology suspicion score. Because PI-RADS was not designed to directly predict NCCN high-risk versus low/intermediate-risk grouping, we report it as a cohort-specific clinical reference rather than a head-to-head substitute for expert interpretation. PI-RADS achieved an AUC of 0.839 (95% CI: 0.726–0.930), comparable to CMOT-Fusion (AUC 0.824). On a paired bootstrap analysis, the AUC difference between CMOT-Fusion and PI-RADS was small and not statistically meaningful (ΔAUC (CMOT–PI-RADS) = -0.015; 95% CI: -0.168 to 0.136). At a common clinical operating point of PI-RADS ≥ 4, the sensitivity and specificity were 0.878 and 0.444, respectively (TP/FP/TN/FN = 36/5/4/5). At PI-RADS ≥ 5, specificity increased to 1.000 with sensitivity 0.634 (TP/FP/TN/FN = 26/0/9/15). To translate AUC into operating-point trade-offs for our model, we additionally selected CMOT-Fusion probability thresholds *post-hoc* on this same test cohort to match these PI-RADS sensitivities (thresholds 0.244910 for sensitivity 0.878 and 0.617629 for sensitivity 0.634), yielding specificity 0.444 (TP/FP/TN/FN = 36/5/4/5) and specificity 1.000 (TP/FP/TN/FN = 26/0/9/15), respectively. We emphasize that this analysis is not a head-to-head reader study; rather, it provides a cohort-specific reference to interpret the model’s discrimination relative to routine clinical scoring.

Comparison Across Fusion Strategies. Examining fusion strategies specifically, CMOT-Fusion outperformed all alternatives in AUC: late fusion approaches (Voting Ensemble (ResNet): 0.772 ± 0.010), intermediate fusion methods (Feature Concatenation (ViT): 0.750 ± 0.028), and attention-based techniques (Cross-Attention (ViT): 0.751 ± 0.018). The performance of single-modality models was comparable between T2WI-ResNet (0.761 ± 0.018) and ADC-ResNet (0.757 ± 0.003), suggesting that the advantages of CMOT-Fusion stem from its effective integration of complementary information rather than from an imbalance in modality-specific predictive power.

### 3.2 Contribution of key model components

To isolate the contributions of the learned cost function and the Optimal Transport mechanism within CMOT-Fusion, we conducted an analysis by comparing the full model against two variants where key components were removed:

1. *No OT*: This variant removes the entire Optimal Transport fusion mechanism, substituting it with simple element-wise addition of the normalized T2WI and ADC features. This evaluates the benefit of the OT-based fusion itself.
2. *Fixed Cost*: This variant retains the OT framework but replaces the learned cost network *c_θ_* with a standard Euclidean distance metric ( $C_{ij}=|\left|f_{T2,norm}^{\left(i\right)}-f_{ADC,norm}^{\left(j\right)}\right|{|}_{2}$). This assesses the value of learning a task-specific cost function.

**Table 4** summarizes the performance of these variants on the internal dataset, with a visual comparison in **Figure 2b**. The full CMOT-Fusion model outperformed both variants. The complete model achieved an AUC of 0.849 ± 0.034, compared to 0.802 ± 0.026 for the “No OT” variant and 0.807 ± 0.030 for the “Fixed Cost” variant. To support robust interpretation, we emphasize the magnitude and consistency of these performance differences across folds, and when formal inference is required we use two-sided, paired patient-level comparisons on pooled out-of-fold predictions rather than relying on fold-level hypothesis tests with only five splits.

**Table 4:** Ablation study results on the internal dataset.

Method	AUC	Accuracy	Sensitivity	Specificity
CMOT-Fusion (Full)	**0.849 ± 0.034**	**0.804 ± 0.032**	**0.755 ± 0.081**	**0.869 ± 0.034**
No OT (Addition)	0.802 ± 0.026	0.760 ± 0.038	0.694 ± 0.143	0.852 ± 0.119
Fixed Cost (Euclidean)	0.807 ± 0.030	0.768 ± 0.027	0.713 ± 0.083	0.841 ± 0.082

Results reported as mean ± standard deviation across 5-fold cross-validation. Bold values indicate the best performance. When formal comparisons are performed, we use two-sided paired patient-level tests on pooled out-of-fold predictions and report effect-size confidence intervals rather than relying on one-sided fold-level tests with only five splits.

These results demonstrate two key findings. First, the principled correspondence finding via Optimal Transport provides substantial benefits over simple feature addition, confirming the value of modeling cross-modal relationships explicitly. Second, the data-driven learning of transport costs specific to the T2WI-ADC fusion task significantly outperforms standard geometric distance metrics, validating our hypothesis that modality-specific correspondence criteria are essential for effective fusion.

### 3.3 Model robustness to parameter selection

We investigated the sensitivity of CMOT-Fusion to two key hyperparameters: the entropic regularization strength *α* in the Sinkhorn algorithm and the number of patches *K*. Experiments were conducted on the internal cross-validation dataset, varying one parameter while keeping the other at its optimal default value (*α* = 0.5*, K* = 32).

The results are presented in **Table 5**. The model achieved the highest mean AUC with the default parameters (*α* = 0.5 *,K* = 32). While variations in *α* (to 0.001, 0.01, 1.0) and *K* (to 16, 64) resulted in slightly lower mean AUCs, we interpret these differences as modest and focus on the observed effect sizes and stability across folds rather than relying on one-sided fold-level hypothesis testing with only five splits.

**Table 5:** Parameter sensitivity analysis on the internal dataset.

Parameter value	Mean AUC ± Std
Entropic Regularization Strength (α)	
0.001	0.838 ± 0.045
0.01	0.810 ± 0.046
**0.5 (Default)**	0.849 ± 0.034
1.0	0.827 ± 0.053
Number of Patches (K)	
16	0.828 ± 0.022
**32 (Default)**	0.849 ± 0.034
64	0.826 ± 0.038

Results reported as mean ± standard deviation across 5-fold cross-validation. Bold values indicate the default parameters used for CMOT-Fusion.

This analysis indicates that while our chosen defaults provide the best observed performance, the model is robust to moderate changes in these key parameters, suggesting good stability for potential clinical application.

### 3.4 Illustrative examples of the fusion mechanism

**Figure 8:**
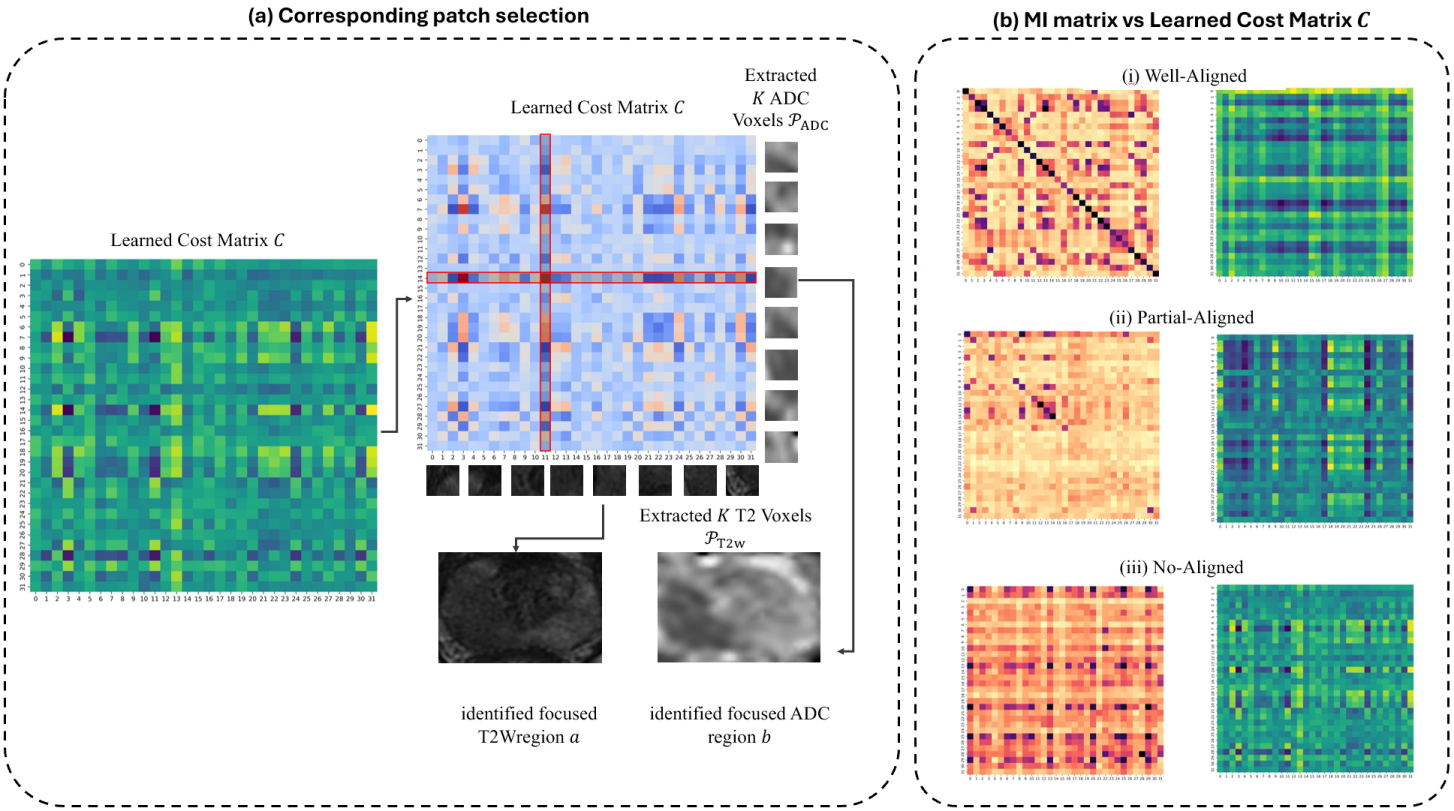
Qualitative illustration of the learned cost matrix’s role in CMOT-Fusion. **(a)** Corresponding patch selection: The learned cost matrix *C* (left) quantifies correspondence costs between T2WI and ADC patch features. A specific entry *C_ij_*in this matrix (e.g., at the intersection of a highlighted row and column in the central heatmap) reflects the cost of associating the *i*-th ADC patch feature (from ‘Extracted K ADC Voxels’, *P_ADC_*) with the *j*-th T2WI patch feature (from ‘Extracted K T2 Voxels’, *P_T_*_2_*_WI_*). Low costs can guide the identification of diagnostically relevant pairs, exemplified by ‘identified focused T2WIregion a’ and ‘identified focused ADC region b’. These regions are depicted as raw image patches (potentially showing cancerous tissue) for illustrative purposes; the Optimal Transport mechanism itself operates on the high-dimensional feature embeddings extracted from such patches, not the raw image data. **(b)** MI matrix vs. Learned Cost Matrix *C*: Comparison between Mutual Information (MI) matrices (left of each pair), reflecting intensity-based patch similarity, and the learned cost matrices *C* (right of each pair) for three scenarios: (i) Well-Aligned, (ii) Partial-Aligned, and (iii) No-Aligned. The learned cost matrix *C* often differs significantly from the MI matrix, demonstrating its ability to capture complex, task-specific relationships that may not correspond to direct spatial alignment or simple intensity correlations, thereby enabling more effective feature fusion.

To gain insight into the fusion mechanism, we visualized the relationships learned by CMOT-Fusion. **Figure 8a** illustrates the patch selection process and how learned costs can identify corresponding diagnostic regions. **Figure 8b** contrasts the learned cost matrix with a standard intensity-based similarity metric (Mutual Information) across different alignment scenarios.

The Mutual Information (MI) matrix, derived from raw patch intensities, reflects spatial correlation and often shows diagonal dominance in well-aligned cases. In contrast, the learned cost matrix *C* computed by the CostNet (*c_θ_*) frequently departs from purely spatial metrics. Low costs in *C* may appear along the diagonal (indicating good alignment is functionally relevant) or at specific off-diagonal locations (correcting for misalignment). Crucially, *C* can also exhibit horizontal or vertical structures, where a single patch from one modality has low association cost with multiple patches from the other. This suggests the network identifies functionally critical features (e.g., a key tumor characteristic in one patch) and learns to associate them broadly with relevant regions in the other modality, prioritizing task-specific utility over strict geometric correspondence.

This learned functional cost structure is a key advantage of CMOT-Fusion. It guides the Optimal Transport solver to establish a nuanced, data-driven correspondence for fusing complementary cross-modal information effectively. By learning a task-specific cost function rather than relying on fixed geometric criteria, the model can achieve robust performance even with misaligned clinical data.

## 4 Discussion

In this study, we address the critical clinical challenge of inter-sequence misalignment in prostate mpMRI, a common problem that compromises diagnostic accuracy. While radiologists attempt to compensate via subjective cognitive fusion, this process is variable and difficult to standardize. We introduce CMOT-Fusion, a framework that directly learns to fuse unregistered T2WI and ADC sequences, achieving a statistically significant improvement in prostate cancer risk stratification (AUC 0.849 internal, 0.824 independent test; **Table 3**). Our work provides a robust solution to real-world data imperfections, paving the way for more reliable automated diagnostic tools.

The current diagnostic pathway hinges on the Prostate Imaging-Reporting and Data System (PI-RADS), but its reliance on the manual co-registration of T2WI and ADC images contributes to significant inter-reader variability (29). Early attempts to automate this process with radiomics were often brittle, requiring precise segmentations that are themselves challenging (30). More recent deep learning models have shown promise but typically circumvent the core problem by assuming perfect image registration, either through curated datasets or fallible preprocessing steps, limiting their real-world utility (9, 31–33). Our work confronts this challenge directly. Instead of enforcing a rigid geometric alignment, CMOT-Fusion learns a task-specific transport cost to establish a functional correspondence between modalities. This allows it to identify diagnostically relevant pairings even in misaligned data, overcoming a primary weakness of prior methods (34).

The clinical implications of improving this foundational imaging analysis are substantial. Uncertainty at the PI-RADS assessment stage directly propagates to downstream patient management, which is guided by NCCN risk stratification. By providing a more accurate and objective risk score that is robust to misalignment, CMOT-Fusion can increase diagnostic confidence and provide a more stable basis for these crucial NCCN categorizations. This, in turn, informs high-stakes decisions, such as the choice between active surveillance and definitive treatment. Ultimately, by enhancing non-invasive risk assessment, our approach helps to better select patients for biopsy, building on the paradigm established by landmark trials to reduce unnecessary invasive procedures (35, 36). Furthermore, by obviating the need for time-consuming mental co-registration, the framework could streamline the clinical workflow.

Despite the demonstrated robustness, CMOT-Fusion remains subject to limitations intrinsic to routine mpMRI acquisition. In particular, ADC derived from echo-planar DWI can be severely degraded by susceptibility distortion (e.g., rectal gas), motion, and low signal-to-noise ratio, yielding regions where diffusion information is unreliable or non-diagnostic. In such cases, the learned transport plan may become diffuse (approaching near-uniform weights) or may form spurious correspondences driven by artifact-related features rather than true tissue properties, which can degrade risk stratification. In addition, very small lesions (e.g.,< 5 mm) and lesions with subtle diffusion restriction may be underrepresented under fixed patch sizes and finite sampling, especially when lesions lie near patch boundaries or are affected by partial-volume effects, although overlapping sampling partially mitigates this. These observations motivate future work on (i) automated image-quality and artifact detection for DWI/ADC with uncertainty-aware fusion, and (ii) multi-scale or lesion-aware patching strategies that improve representation of small or heterogeneous tumors.

This study has several limitations. The findings are based on data from a single institution with a small independent temporal test cohort (n=50) and a high test-set high-risk prevalence (82.0%); therefore, the results should be interpreted as exploratory, and a large-scale, multi-center prospective study is required to validate generalizability across patient populations, prevalences, and imaging hardware (37). In addition, our preprocessing relies on automated prostate gland segmentation to define the normalization region and cropping ROI; segmentation errors could propagate to downstream classification performance. Because the segmentation tool is commercial and its training data are proprietary, we cannot report cohort-specific segmentation accuracy, and we did not perform a sensitivity analysis comparing manual versus automated masks. Finally, although our contribution is to demonstrate strong risk stratification performance without requiring explicit inter-sequence registration, we did not evaluate a “registration-then-fusion” baseline (e.g., rigid + deformable registration using ANTs (38) or elastix (39). A fair comparison would require careful parameterization and validation under DWI/ADC distortion (including quality control and failure handling) while preserving comparable ROI/patch extraction after warping; we identify this benchmark as an important direction for future work. Additionally, we did not quantify the magnitude of inter-sequence misalignment (e.g., millimeters of displacement) between T2WI and ADC in this cohort. Establishing a ground truth for such measurements would require manual non-rigid alignment and/or expert landmark annotation, both of which are subjective and reader-dependent. Accordingly, our evidence for misalignment robustness is indirect: we demonstrate consistent downstream improvements without explicit registration under routine clinical acquisition conditions. Future work should quantify misalignment severity using reproducible surrogates (e.g., landmark-based displacement, gland-mask overlap/contour distances after rigid alignment, or deformation fields from a validated registration protocol) and evaluate performance stratified by misalignment severity to more directly attribute gains to registration-free correspondence learning. While we demonstrate improved diagnostic accuracy, a formal reader study is necessary to quantify the impact of CMOT-Fusion on clinical decision-making and patient outcomes, following established guidelines for AI evaluation (40). Future work will focus on this validation, as well as exploring the interpretability of the learned transport plans as a novel form of explainable AI (XAI) to build clinical trust (41).

## 5 Conclusion

In conclusion, CMOT-Fusion provides a novel and effective solution to the common clinical problem of inter-sequence misalignment in prostate mpMRI. By learning to identify and fuse the most functionally relevant information across modalities, our framework achieves a new level of robustness and accuracy. This work demonstrates the significant potential of learned, correspondence-based fusion to enhance the reliability of medical imaging diagnostics, paving the way for more powerful AI tools in clinical practice.

## Data Availability

The raw data supporting the conclusions of this article will be made available by the authors, without undue reservation.
