# Supplementary material for: Robust prostate cancer risk stratification from unregistered mpMRI via learned cross-modal correspondence
Source: Front Oncol. 2026 Jan 29;15:1700447. doi: 10.3389/fonc.2025.1700447 (PMC12896216; doi:10.3389/fonc.2025.1700447)
Supplement: Supplementary file 1 [file DataSheet1.docx]

**Supplementary Materials**

**Generative AI prompts used in manuscript preparation**

Language refinement prompt: "Please refine the following academic text for clarity, concision, grammar, and journal-appropriate scientific tone without altering the meaning, data, analyses, or conclusions. Return only the revised text."

Language assembly prompt: "Please assemble the following fragmented notes/sentences into a coherent, grammatically correct academic paragraph suitable for a medical imaging journal, preserving the authors' intended meaning and not creating new facts or interpretations."
